# Considerations for the Surgical Management of Thoracic Cancers During the COVID-19 Pandemic: Rational Strategies for Thoracic Surgeons

**DOI:** 10.3389/fsurg.2022.742007

**Published:** 2022-05-09

**Authors:** Jiahao Zhang, Yichao Han, Yajie Zhang, Dong Dong, Yuqin Cao, Xiang Chen, Hecheng Li

**Affiliations:** Department of Thoracic Surgery, Ruijin Hospital, Shanghai Jiao Tong University School of Medicine, Shanghai, China

**Keywords:** COVID-19, early-stage lung cancer, esophageal cancer, ground glass opacities, recommendation, prevention of nosocomial transmission in hospitals, thoracic surgeons, surgical treatment

## Abstract

**Objective:**

The novel Coronavirus Disease 2019 (COVID-19) has resulted in a global health crisis since first case was identified in December 2019. As the pandemic continues to strain global public health systems, elective surgeries for thoracic cancer, such as early-stage lung cancer and esophageal cancer (EC), have been postponed due to a shortage of medical resources and the risk of nosocomial transmission. This review is aimed to discuss the influence of COVID-19 on thoracic surgical practice, prevention of nosocomial transmission during the pandemic, and propose modifications to the standard practices in the surgical management of different thoracic cancer.

**Methods:**

A literature search of PubMed, Medline, and Google Scholar was performed for articles focusing on COVID-19, early-stage lung cancer, and EC prior to 1 July 2021. The evidence from articles was combined with our data and experience.

**Results:**

We review the challenges in the management of different thoracic cancer from the perspectives of thoracic surgeons and propose rational strategies for the diagnosis and treatment of early-stage lung cancer and EC during the COVID-19 pandemic.

**Conclusions:**

During the COVID-19 pandemic, the optimization of hospital systems and medical resources is to fight against COVID-19. Indolent early lung cancers, such as pure ground-glass nodules/opacities (GGOs), can be postponed with a lower risk of progression, while selective surgeries of more biologically aggressive tumors should be prioritized. As for EC, we recommend immediate or prioritized surgeries for patients with stage Ib or more advanced stage and patients after neoadjuvant therapy. Routine COVID-19 screening should be performed preoperatively before thoracic surgeries. Prevention of nosocomial transmission by providing appropriate personal protective equipment (PPE), such as N-95 respirator masks with eye protection to healthcare workers, is necessary.

## Introduction

Coronavirus Disease-2019 (COVID-19) is caused by infection of the novel coronavirus severe acute respiratory syndrome coronavirus 2 (SARS-CoV-2) virus, which has widely spread worldwide and resulted in over 1.8 hundred million infection cases and 3,930,000 deaths worldwide (1). The SARS-CoV-2 virus was first identified in the city of Wuhan, China in 2019. SARS-CoV-2 is a highly contagious pathogen transmitted from human to human through exposure to respiratory fluids carrying an infectious virus, and the average reproductive ratio (R_0_) for SARS-CoV-2 has been found between 1.4 and 2.5 transmissions by one initial infection (2). The incubation period of SARS-CoV-2 is found most likely to be ≤14 days (median, 5 days) and the average mortality rate of COVID-19 is 3.6% (3) whereas mortality for critical cases reaches up to 60.5% (4). Patients with symptoms can develop fever, cough, fatigue, expectoration, and anhelation as the most common clinical manifestations after being infected by SARS-CoV-2. The elderly or those with previous and current existing chronic medical conditions may suffer from relatively severe viral pneumonia, and then some of these patients may progress to SARS and eventually death (5). Moreover, there are patients who are not yet experiencing onset of symptoms (presymptomatic) and patients without typical symptoms (asymptomatic) of COVID-19 disease (6), which increases the risk of human to human transmission in population.

As the COVID-19 pandemic constantly strains the health systems in almost every nation, interfering with the organization of normal healthcare delivery in severe epidemic areas, it has temporarily postponed a large proportion of elective surgeries for early-stage lung cancers and other thoracic cancers worldwide. The delay of lung cancer resections brings the risk of disease progression. Further, social distancing policies have affected timely evaluation for the initial diagnosis and follow-ups of suspected non-small cell lung cancer (NSCLC) and esophageal cancer (EC) in outpatient clinics. Therefore, based on the case load of COVID-19 in a certain area, the surgical treatment of thoracic cancers needs to overcome these difficulties with appropriate modifications to the standard practices by surgeons and healthcare workers, and certain measures are supposed to be adapted for minimalizing the risk of transmission. This review aims to share and discuss the latest clinical evidence and provide a framework and proposal for modifications to the current practices of managing early-stage NSCLC and EC that contributes to the promptly surgical treatment and safety of patients, and protection for healthcare providers during the ongoing pandemic, from a thoracic surgeon's perspective.

## Preventing Nosocomial Transmission in Thoracic Surgery: Preoperative Screening and Personal Protection

Previous reports have shown that the infection of SARS-CoV-2 in the perioperative period is associated with a relatively poor prognosis for patients who underwent thoracic surgical procedures, and healthcare workers are vulnerable to transmission from infected patients prior to appropriate personal protective equipment (PPE) utilization (7, 8). Thus, preventing and breaking the chains of nosocomial transmission during the pandemic should be the priority of concerns in the thoracic surgery department.

The transmission of the SARS-CoV-2 virus from infected individuals to other people is mainly by inhaling aerosolized droplets containing the virus and direct contact with infected patients. There is no evidence of airborne transmission through aerosols of SARS-CoV-2 up to the present. The smallest aerosolized droplets containing SARS-CoV-2 have been found to be as small as 5 μm, and those droplets can be produced during nearly all procedures concerning the bronchus and upper areo-digestive tract, such as bronchoscopy, endotracheal intubation, endobronchial ultrasound (EBUS), upper endoscopy, or surgeries, involving the bronchus and lung that include radical resection of lung cancer (such as lobectomy, pneumonectomy, segmentectomy, and wedge resections) (2). Surgical damage to the bronchial, upper areo-digestive tract mucosa, or lung parenchyma might lead to aerosolization of the droplets containing the SARS-CoV-2 virus and increase the risk of transmission in the operating room. Similarly, Mckeown and Ivor-Lewis esophagectomy also involves a thoracic phase surgery, which increases the risk of infection.

According to WHO and Central Disease Control, appropriate personal protection equipment is recommended for all medical staff during thoracic surgeries (9). Notably, standard precautions may not work as the optimal protection in the operating room (OR) during procedures with high risks of aerosol-generating as the aerosolized viral particles may not be effectively filtered by a standard droplet mask for their 5 μm diameter. For the optimal personal protection of thoracic surgeons, anesthetists, and other hospital staff inside the operating room, the application of N-95 respirator masks for all aerosol/droplet-generating procedures regardless of the local COVID-19 pandemic status is always recommended (2), and eye-protective goggles for preventing direct liquid splashing should be considered for surgeons and scrub nurses.

Apart from that, current reports suggest a range of 30–60% (10) proportion of patients with asymptomatic COVID-19. Given that the number might be greater than previously expected (2), the risk of COVID-19 transmission from patients without symptoms or with only mild symptoms to other uninfected patients or health hospital staff is unknown, which further increases the difficulty to distinguish nosocomial infection from community infection (2). The potential existence of asymptomatic “super spreader” patients also increases the possibility of nosocomial transmission in the ward and operating theater (8). In order to identify these asymptomatic patients in advance, a routine preoperative screening of SARS-CoV-2 by sampling from nasal or nasopharyngeal swabs and testing the virus RNA by real-time reverse transcription polymerase chain reaction (RT-PCR) is indispensable for alleviating the risk for intraoperative and perioperative nosocomial transmission by asymptomatic patients. Although the accuracy and reliability of the test are influenced by a number of factors that include sampling technique, sampling timing, quality of the testing kits, and the evolution of the disease, routine screening of SARS-CoV-2 by RT-PCR for all patients who are admitted or transferred to the thoracic surgery department is recommended for its convenience and relative reliability, especially for patients who are undergoing procedures with a high risk of transmission, such as bronchoscopy or lung cancer surgery. Considering the false negativity of the RT-PCR test, repeat testing should be considered for negative patients with symptoms or patients with contact history.

### Teaching Points

Application of N-95 respirator masks and eye-protective goggles for all aerosol/droplet-generating procedures in the operating room and a routine preoperative screening of SARS-CoV-2 by RT-PCR regardless of the local COVID-19 pandemic status are recommended for alleviating perioperative nosocomial transmission.

## Diagnosing and Surgical Management of Early-Stage NSCLC During the COVID-19 Pandemic

### Comparing and Differentiating COVID-19 From Early-Stage Lung Cancer

During the COVID-19 pandemic, making an accurate diagnosis in asymptomatic patients is more challenging. Computed tomography (CT) scans of patients with COVID-19 without symptoms have sometimes identified related lung lesions (2, 11, 12), and patients with COVID-19 sometimes have presented similarly with early-stage lung cancer on CT scans. It has been reported that most COVID-19 cases are initially featured as patchy or oval ground-glass opacities (GGOs), yet others are featured by consolidative nodules on the chest CT scans, while the progression of virus pneumonia is usually accompanied by the enlargement and consolidation of the GGOs (13, 14). Taking a thorough history, a careful review of imaging, and appropriate testing are required to make an accurate diagnosis. Hereby, we discuss the radiographic similarities and distinctions between early-stage lung cancer and COVID-19 with GGO features that include aspects of spatial location, lesion components, specific radiographic features, and dynamic changes (Table 1).

**Table 1:** Radiological differences between Coronavirus Disease-19 (COVID-19) and early-stage lung cancer with GGO features.

	**COVID-19**	**Early-stage lung cancer**
**Spatial location**		
Number	Multiple	Solitary
Laterality	Bilateral	Unilateral
Location	Peripheral	Peripheral
Involved lobes	2–4	1
Involved Segments	10–12	1
**Component**	Pure GGO and mixed GGO	Pure GGO and mixed GGO
**Form**	Patchy	Oval
**Radiographic features**	Interlobular septal thickening	Lobulation
	Air bronchogram	Spiculation
	Crazy-paving pattern	Notches
	Reticular pattern	Vascular convergence
	Halo sign	Pleural indentation
**Temporal progression**		
Progression rate	Rapid	Slow
Disease period	2-6 weeks	VDT of pure GGO: 813 days VDT of mixed GGO: 457 days
Changes on CT	GGOs grow and resolve	GGOs grow
	Lung involvement increases	Increasing or new solid component
	Consolidation	

*GGO, ground-glass opacity; VDT, volume doubling time*.

Solitary GGO predominates in early-stage lung cancer, and morbidity of multiple GGOs has been increasing gradually (15). Multiple GGOs can be either unilateral or bilateral, with no obvious preference (16, 17). In contrast, GGO lesions for COVID-19 are usually multiple and located bilaterally (14). Pulmonary involvement is generally more diffuse in patients with COVID-19 and affects more lobes and segments than early lung cancer patients.

Ground-glass opacities are radiologically categorized into pure GGOs, which have no solid component, and part-solid, or mixed GGOs, which contain both a GGO and a solid component (18). Pure GGOs and mixed GGOs are both common on CT scans in COVID-19. In a multicenter retrospective study, 86% of 101 COVID-19 patients were presented with pure GGOs on CT while 64% showed mixed GGOs (19), data from our institution also showed that 100 and 63.6% of 11 patients with COVID-19 had pure GGOs and mixed GGO, respectively (20). On the contrast, although the proportion of pure or mixed GGO in early lung cancer is unclear, a large volume study has shown that 73 cases in 82 pure GGOs and 79 cases in 102 mixed GGOs are confirmed for lung cancers (21, 22). Thus, GGOs in early-stage lung cancer and COVID-19 do not have a predilection toward pure or mixed GGOs. Besides, the patchy form was much more frequent than the oval form in patients with COVID-19 (23), while early lung cancer was more frequently presented as the round or oval form (24).

In addition, studies of patients with COVID-19 discovered that ill-defined margins and air bronchograms are the most common imaging features observed except for GGO, along with thickening of interlobular septal and crazy-paving pattern (11). Reticular patterns, halo signs, air bronchograms, and fibrous stripes can also be seen (23, 25).

The clinical course on CT scans in the two disease entities over time is also significantly different. As an acute disease, COVID-19 has a rapid progression over a period of 2 and 3–6 weeks for mild and severe cases, respectively. Four stages of pneumonia progression on chest CT scan were proposed as the early stage (0–4 days after initial diagnosis) featured by GGOs, progressive stage (5–8 days) featured by increasing total CT score, peak stage (10–13 days) featured by consolidation, and absorption stage (≥14 days) at last (26). During the progressive stage, GGOs grew larger and lung involvement was increased, with the onset of a crazy-paving pattern and consolidation, with the whole process of progression usually within weeks (26). On the contrast, early-stage lung cancers with GGO features are regarded as indolent tumors. These indolent or slow-growing GGOs are often defined by volume doubling time (VDT), i.e., the time required for an enlarging tumor to double its volume. A screening program for early lung cancer in Japan, where 78% were adenocarcinomas, reported mean VDTs of 813 and 457 days for pure GGOs and mixed GGOs, respectively (27), which is distinctly longer than COVID-19 progression. Therefore, although the initial imaging profiles may be similar, the dynamic development of CT findings in early-stage lung cancer is much slower than COVID-19, which can also resolve over time in contrast to malignancy, which emphasizes the follow-up CT imaging for differentiation.

Taken together, the imaging characteristics of COVID-19 are similar but partially different from early-stage lung cancer presenting as GGOs, which can be recognized by thorough and meticulous examination of CT scan on the first diagnosis in clinics. However, to achieve a reliable diagnosis of early lung cancer and to differentiate it from COVID-19, imaging profiles of suspected patients should be sufficiently understood and comprehensively analyzed combined with clinical manifestations, epidemiological history especially contact history with infectious sources, laboratory tests, pathogen screening, CT re-examination, and pathological analysis acquired from surgical treatment (28).

### Indication and Triage of Surgical Intervention for Early-Stage Lung Cancers During the COVID-19 Pandemic

The decision on treating suspected early-stage lung cancers, generally by pulmonary resection, depends on the risk evaluation through chest CT imaging. However, the risk of disease progression needs to be balanced with the risk of COVID-19 infection during this COVID-19 pandemic. Prompt surgical treatment for lung cancer has also been challenged by restricted operating room capacity. The optimal treatment decisions for thoracic surgeons during the pandemic can be made by proposing a triage system for NSCLC cases, arranging surgical resection at the appropriate time without significantly increasing the risk of cancer progression.

The American College of Surgeons (ACS) had released a well-considered guideline in late March 2020 for triaging cancer surgery during the pandemic. This guideline outlined the general principles of selection and prioritization for thoracic surgery cases in three outbreak phases of COVID-19 (29). According to ACS and Thoracic Surgery Outcomes Research Network, three phases are proposed according to the COVID-19 trajectory within the hospital and limitations in hospital resources: semi-urgent setting, urgent setting, and fully urgent setting. Elective surgeries, such as resection of lung GGOs, should be restricted to the semi-urgent situation when the volume of patients with COVID-19 is limited and hospital resources are not in scarcity. An analysis of database (30) has found that surgical delay for more than 8 weeks was an independent risk factor related to the progression of operable NSCLC (30). Therefore, the period of delay in early NSCLC patients waiting to have surgery should be less than 2 months to avoid increasing the risk of disease progression (2). Accordingly, surgery for early-stage lung cancer should become one of the top priorities in surgical cases scheduling, especially when oncological results and survival are influenced by progression caused by overlong delay. If necessary, consider those patients as second priority surgical cases if the delay is estimated to be no more than 2 or 3 months (2). According to the ACS Guidelines on Triaging Thoracic Patients, resection should be restricted to the semi-urgent phase and limited to predominantly solid (>50%) or presumed lung cancers >2 cm or nodal positive disease (29).

As for the specific indication for surgical intervention, the National Comprehensive Cancer Network (NCCN) Guidelines have recommended 6 mm as the cutoff values for the evaluation of incidental nodules (31). Intervention should be considered if the nodules develop a solid or invasive component, although the relatively lower risk of progression of an indolent, slow-growing nodule should be balanced with the risks and resource constraints that the local hospital system is facing due to COVID-19 (31).

Non-small cell lung cancer consists of a broad spectrum of different cancer types with growth patterns and grades of malignancy based on their location, image characteristics, pathology type, invasiveness, and tumor differentiation. Interestingly, the tumor's standardized uptake value (SUVmax) from positron emission tomography-Computed tomography (PET-CT) may be related to a higher rate of advanced tumor features, such as mediastinal lymph node metastasis, pleural and pericardial invasion found at operation, and a higher possibility of postoperative pathologic upstaging, but not statistically significantly (12.0 vs. 9.4, *p* = 0.08) (32). Therefore, SUVmax may also be regarded as a factor for deciding the priority of early-stage lung cancer surgeries. Apart from that, a comprehensive triaging system should also be created with consideration of tumor staging, histology, and imaging features, which might indicate invasiveness, to prioritize the more malignant, aggressively behaving tumors for surgical intervention, and postpone the surgery for relatively indolent lesions.

#### Teaching Points

To accurately differentiate early lung cancer from COVID-19, sufficient understanding and comprehensive analysis of imaging profiles of suspected patients should be combined with clinical manifestations, epidemiological history, laboratory tests, pathogen screening, CT re-examination, and pathological analysis is recommended. A comprehensive triaging system considering tumor staging, histology, and imaging features indicating invasiveness along with SUVmax from PET-CT should be created to prioritize the more malignant, aggressively behaving tumors.

### Timing and Consideration of Surgical Management for EC During the COVID-19 Pandemic

Esophageal cancer is an aggressive thoracic malignancy, and the majority of EC patients typically requires neoadjuvant therapy before surgical treatment, except in the earliest stage patients. A typical esophagectomy usually consists of two (Ivor-Lewis) or three (Mckeown) phases, both include a transthoracic phase, except transhiatal esophagectomy (THE). Transthoracic esophagectomy involves dissociation and dissection of the thoracic esophagus, mediastinal lymph node dissection, and esophageal-tubular stomach anastomosis, which facilitates the generation of aerosolized droplets and increases the risk of postoperative pulmonary complications and SARS-CoV-2 infection. Besides, esophagectomies are highly complicated and demanding procedures with a median operation time of 6 h for transthoracic esophagectomy surgeries (33), surgeons thus limited operating room capacity and medical supplies that include qualified PPE also contribute to the delay of a timely esophagectomy for patients. These factors have deeply impacted the timely scheduling of EC surgeries during the COVID-19 pandemic.

In the guidelines published by ACS for the triage of cancer surgery, immediate surgery have been recommended for patients with stage Ib or more advanced stage EC, and EC patients who have finished preoperative neoadjuvant therapy, while considering endoscopic resection (ER) as a minimally invasive treatment through natural orifice for amenable early-stage Ia/b EC patients has also been suggested (34). However, ER treatment should be decided by completely assessing the possibility of paraoesophageal lymph node metastasis, which rarely occurs in carcinoma *in situ* or early-stage EC within the lamina propria. The indication for ER is the same for early esophageal adenocarcinoma and squamous cell carcinoma patients (35).

Esophagectomy plus lymphadenectomy is recommended to be the standard treatment for resectable stage I (cT1bN0) esophageal squamous cell carcinoma (SCC) patients (35). For patients with EC in this stage, minimal evidence exists by now has investigated the influence of surgical delay on the patients' oncological outcomes, but it does suggest that prolonged waiting time before esophagectomy for over 50 days might be related to a decreased survival, thus surgical resection should not be delayed as much as possible (36). Raman (37) found that for patients with T1N0M0 esophageal AC who underwent esophagectomy without preoperative therapy, a delay of less than 50 days led to improved survival (hazard ratio [HR] = 0.991). On the other hand, time to surgery longer than 100 days was related to worse survival (HR = 1.003) and an increased positive margin rate during surgery (odds ratio [OR] = 1.01) (37). One study by Visser (38) also found that overall survival (OS) was worse with each additional week of surgical delay for patients acquiring primary surgeries, but the waiting time (<8 weeks or ≥ 8 weeks) had no statistically significant impact on disease-free survival (DFS) (HR = 1.03, *p* = 0.443) or OS (HR = 1.06, *p* = 0.108), and it did not significantly influence DFS (*p* = 0.884) or OS (*p* = 0.374) for patients treated with neoadjuvant therapy followed by surgery or primary surgery in total (38).

Apart from primary surgeries, most of the currently existing evidence has investigated the impact of time to surgery after finishing neoadjuvant therapy. The results from the studies that focus on surgery delays after neoadjuvant therapy are mixed. Ranney et al. (39) found that for patients with stages II and III esophageal AC who accepted neoadjuvant chemoradiotherapy (nCRT) and subsequent esophagectomy surgery, long-interval (the time between nCRT and surgery over 56 days) patients had a higher rate of pathologic downstaging (OR = 1.38, *p* = 0.04), but there was no significant difference in resection margin positivity when compared with short-interval (no more than 56 days) patients (OR = 0.91, *p* = 0.69). They also found worse OS in the long-interval subgroup (HR = 1.44, *p* < 0.001). Besides, there was a difference in the 30-day postoperative mortality rate but the difference was statistically insignificant (OR = 1.56, *p* = 0.12) (39). In addition, a meta-analysis evaluating the association between short- and long-term outcomes of EC treatment and the length of the interval between neoadjuvant chemoradiation therapy and surgery indicates that an interval longer than 7–8 weeks has improved the pathologic complete response (pCR) rate [relative risk (RR) = 1.13, *p* = 0.001] but decreased 2- and 5-year OS (RR = 0.94, *p* = 0.002; RR = 0.88, *p* = 0.0009, respectively) (40). A longer interval was also associated with higher 30-day surgical-related perioperative mortality (RR = 1.51, *p* = 0.0006) (40). Therefore, the optimal interval to surgery following neoadjuvant therapy is possibly approximately 6–8 weeks (36). The interval can be slightly prolonged according to the severity of the epidemic and medical surge capacity in different regions, but we should exert efforts to avoid surgical delay for more than 8 weeks.

Another important note to be considered for patients with EC during the COVID-19 pandemic is that a few studies have revealed a possible equivalence of definitive chemoradiotherapy (dCRT) to trimodality therapy (TMT) that includes nCRT and esophagectomy in OS of loco-regionally advanced, resectable patients with EC (41). In meta-analyses that include 26,917 resectable, curatively treated patients with EC from 33 studies, when analyzing by equal patient groups, there was no significant difference between dCRT and TMT – 3- and 5-year OS with an RR of 0.83 for 2-year OS, 0.81 for 3-year OS, and 0.63 for 5-year OS, respectively (41). In the situation of severe medical resources shortage, especially OR capacity scarcity due to the COVID-19 pandemic, the possibility of dCRT as an alternative for nCRT followed by esophagectomy should be considered.

#### Teaching Points

Timely scheduling of EC surgeries during COVID-19 is important for improving survival. Immediate surgery should be considered for patients with stage Ib or more advanced stage EC and EC patients who have finished preoperative neoadjuvant therapy. The optimal interval to surgery following neoadjuvant therapy is possibly approximately 6–8 weeks, which can be slightly prolonged but surgical delay for more than 8 weeks should be avoided as much as possible.

## Conclusion

During the COVID-19 pandemic, the overall priority is patient safety and optimization of hospital systems to fight against COVID-19. Radiological evaluation of lung GGOs should receive adequate attention for suspected early-stage lung cancer patients, and surgeries for lung tumors behaving indolently, such as pure GGO tumors, can likely be postponed, while selective cases that include resection of more biologically aggressive tumors should be prioritized. As for EC treatment during the pandemic, immediate or prioritized surgery for stage Ib or greater operable EC and for patients after neoadjuvant therapy is recommended, and the delay of surgery should not exceed 8 weeks. ER can be considered for amenable early-stage Ia/b EC without risk of lymph node metastasis. dCRT might be considered as an alternative for nCRT followed by esophagectomy for resectable loco-regionally advanced patients with EC in certain circumstances. Preoperative COVID-19 screening is recommended to be routinely and repetitively performed before undergoing any thoracic surgeries. Surgeons should use proper PPE that includes N-95 respirators with eye protection during surgery or direct contact with suspected infected patients.

## Author Contributions

All authors listed have made a substantial, direct, and intellectual contribution to the work and approved it for publication.

## Funding

This study was supported by National Natural Science Foundation of China (82072557 and 81871882), National Key Research and Development Program of China (2021YFC2500900), Shanghai Municipal Commission of Health and Family Planning Outstanding Academic Leaders Training Program (2017BR055), and Shanghai Municipal Education Commission-Gaofeng Clinical Medicine Grant (20172005).

## Conflict of Interest

The authors declare that the research was conducted in the absence of any commercial or financial relationships that could be construed as a potential conflict of interest.

## Publisher's Note

All claims expressed in this article are solely those of the authors and do not necessarily represent those of their affiliated organizations, or those of the publisher, the editors and the reviewers. Any product that may be evaluated in this article, or claim that may be made by its manufacturer, is not guaranteed or endorsed by the publisher.

## Abbreviations

COVID-19: Coronavirus disease 2019
SARS-CoV-2: Severe acute respiratory syndrome coronavirus
GGOs: Ground-glass opacities
NSCLC: Non-small cell lung cancer
RT-PCR: Real-time polymerase chain reaction
CT: Computed tomography
LDCT: Low-dose CT
VDT: Volume doubling time
NCCN: the National Comprehensive Cancer Network
ACS: the American College of Surgeons
CDC: Centers for Disease Control
OR: Operating room
PPE: Personal protective equipment
HR: Hazard ratio
OR: Odds ratio
RR: Relative risk
nCRT: Neoadjuvant chemoradiation therapy
EC: Esophageal cancer
SCC: Squamous cell carcinoma
AC: Adenocarcinoma
DFS: Disease-free survival
OS: Overall survival
pCR: Pathologic complete response
ER: Endoscopic resection
dCRT: Definitive chemoradiotherapy
TMT: Trimodality therapy
PET-CT: Positron emission tomography-Computed tomography.
